# Process data of allogeneic ex vivo*-*expanded ABCB5^+^ mesenchymal stromal cells for human use: off-the-shelf GMP-manufactured donor-independent ATMP

**DOI:** 10.1186/s13287-020-01987-y

**Published:** 2020-11-16

**Authors:** Seda Ballikaya, Samar Sadeghi, Elke Niebergall-Roth, Laura Nimtz, Jens Frindert, Alexandra Norrick, Nicole Stemler, Nicole Bauer, Yvonne Rosche, Vanessa Kratzenberg, Julia Pieper, Tina Ficek, Markus H. Frank, Christoph Ganss, Jasmina Esterlechner, Mark A. Kluth

**Affiliations:** 1 TICEBA GmbH, Im Neuenheimer Feld 517, 69120 Heidelberg, Germany; 2 Transplant Research Program, Boston Children’s Hospital, Harvard Medical School, Boston, MA USA; 3 Harvard Stem Cell Institute, Harvard University, Cambridge, MA USA; 4 Department of Dermatology, Brigham and Women’s Hospital, Harvard Medical School, Boston, MA USA; 5 School of Medical and Health Sciences, Edith Cowan University, Perth, Western Australia Australia; 6 RHEACELL GmbH & Co. KG, Im Neuenheimer Feld 517, 69120 Heidelberg, Germany

**Keywords:** Advanced-therapy medicinal product, ABCB5, GMP manufacturing, Mesenchymal stromal cells

## Abstract

**Background:**

Human dermal mesenchymal stromal cells (MSCs) expressing the ATP-binding cassette (ABC) efflux transporter ABCB5 represent an easily accessible MSC population that, based on preclinical and first-in-human data, holds significant promise to treat a broad spectrum of conditions associated not only with skin-related but also systemic inflammatory and/or degenerative processes.

**Methods:**

We have developed a validated Good Manufacturing Practice-compliant expansion and manufacturing process by which ABCB5^+^ MSCs derived from surgical discard skin tissues are processed to an advanced-therapy medicinal product (ATMP) for clinical use. Enrichment for ABCB5^+^ MSCs is achieved in a three-step process involving plastic adherence selection, expansion in a highly efficient MSC-selecting medium, and immunomagnetic isolation of the ABCB5^+^ cells from the mixed culture.

**Results:**

Product Quality Review data covering 324 cell expansions, 728 ABCB5^+^ MSC isolations, 66 ABCB5^+^ MSC batches, and 85 final drug products reveal high process robustness and reproducible, reliable quality of the manufactured cell therapy product.

**Conclusion:**

We have successfully established an expansion and manufacturing process that enables the generation of homogenous ABCB5^+^ MSC populations of proven biological activity manufactured as a standardized, donor-independent, highly pure, and highly functional off-the-shelf available ATMP, which is currently tested in multiple clinical trials.

## Background

While mesenchymal stromal cells (MSCs) for therapeutic use are most frequently obtained from bone marrow, followed by adipose tissue and puerperal tissues such as umbilical cord and placenta, MSCs can be isolated from a far greater variety of human tissues [1–3]. Human skin, representing a particularly easily accessible tissue that offers the use of surgical discard tissue, harbors a highly potent immunomodulatory cell population marked by the plasma membrane-spanning ATP-Binding Cassette Transporter, Subfamily B, Member 5 (ABCB5) [4]. Human skin-derived ABCB5^+^ cells display typical MSC characteristics as defined by the International Society for Cellular Therapy [5], including adherence to plastic surfaces; clonogenicity; expression of CD90, CD73, and CD105; lack of hematopoietic lineage markers CD45, CD34, and CD14; increased osteogenic, adipogenic, and chondrogenic differentiation potential as compared with donor-matched ABCB5^−^ fibroblasts [6]; and trans-differentiation into CD31^+^ endothelial cells [7]. In addition to their (trans-) differentiation potential, ABCB5^+^ MSCs have shown marked immunomodulatory effects through interaction with macrophages [6], neutrophils [8], and regulatory T cells [9], as well as a strong paracrine capacity including adaptive release of vascular endothelial growth factor (VEGF) and interleukin 1 receptor antagonist (IL-1RA) [6, 7].

Covering all three principal mechanisms (i.e., immunomodulation, adaptive secretion of anti-inflammatory and pro-angiogenic biomolecules, and multilineage differentiation) by which MSCs can contribute to inflammation control and tissue repair, skin-derived ABCB5^+^ MSCs offer a broad spectrum of potential therapeutic indications associated not only with skin-related but also systemic inflammatory and/or degenerative processes. In preclinical studies, ABCB5^+^ MSCs delivered significant benefit in mouse models of chronic skin wounds [6], epidermolysis bullosa [10], and liver disease [11]. In a first in-human clinical trial, topically applied ex vivo-expanded ABCB5^+^ MSCs facilitated wound closure of standard therapy-resistant chronic venous ulcers [7].

Aiming at developing human skin-derived ABCB5^+^ MSCs for clinical use as an advanced-therapy medicinal product (ATMP), we have developed and validated a Good Manufacturing Practice (GMP)-compliant ex vivo expansion and manufacturing process to reliably, reproducibly, and efficiently generate highly pure ABCB5^+^ MSCs of proven biological potency that can be instantly manufactured as an off-the-shelf product at a clinical scale. Enrichment for ABCB5^+^ MSCs, which account for roughly 2–3% of the total dermal cell population [6, 12], is achieved in a three-step process involving selection upon plastic adherence, ex vivo cell expansion in a highly efficient MSC-selecting medium, and immunomagnetic isolation of the ABCB5^+^ cells from the mixed culture, making use of an antibody specifically directed against an extracellular loop of the ABCB5 molecule [4]. This product has been tested in a thorough preclinical biodistribution, toxicity, and tumorigenicity study program, which has revealed a favorable safety profile after subcutaneous, intramuscular, and intravenous single- and repeated-dose application [13]. Currently, our product is being assessed in several multicentric national and international clinical trials evaluating its safety and efficacy in various chronic inflammatory and degenerative conditions (Table 1).

**Table 1:** Drug products manufactured from ABCB5^+^ MSCs for use as investigational medicinal products in clinical trials

Product		Clinical trial				Product characteristics			
Name	Number of batches produced (total: 85)	ClinicalTrials.gov identifier	Indication	Application route	Cell dose per application	Composition	Total live cell count	Container	Pack size
allo-APZ2-CVU	30	NCT03257098	Chronic venous ulcer	topical	1 × 10^6^ cells/cm^2^ wound surface	1 × 10^7^ ABCB5^+^ MSCs in 1 ml HRG	dependent on wound size (max. 100 × 10^6^)	1-ml syringe	1–10 syringes (dependent on wound size)
allo-APZ2-DFU	26	NCT03267784	Diabetic foot ulcer	topical	2 × 10^6^ cells/cm^2^ wound surface	1 × 10^7^ ABCB5^+^ MSCs in 1 ml HRG		1-ml syringe	1–10 syringes (dependent on wound size)
allo-APZ2-EB	23	NCT03529877	Epidermolysis bullosa (recessive dystrophic)	intravenous	2 × 10^6^ cells/kg body weight	1 × 10^7^ ABCB5^+^ MSCs in 1 ml HRG	dependent on body weight	10-ml syringe	1–2 syringes (dependent on body weight)
allo-APZ2-ACLF	3	NCT03860155	Acute-on-chronic liver failure	intravenous		1 × 10^7^ ABCB5^+^ MSCs in 1 ml HRG	dependent on body weight	10-ml syringe	1–2 syringes (dependent on body weight)
allo-APZ2-PAOD	3	NCT03339973	Peripheral artery disease	intramuscular	7.5 × 10^6^ cells per injection site (20–30 injection sites, depending on leg length)^a^	1 × 10^7^ ABCB5^+^ MSCs in 1 ml HRG	150 × 10^6^–225 × 10^6^, dependent on leg length	1-ml syringe	20–30 syringes (number equal to the number of injection sites)

*HRG* Ringer’s lactate solution containing human serum albumin and glucose, *MSC* mesenchymal stromal cell

^a^Number of injection sites = 2/3 × lower leg length (defined as distance in cm between the popliteal space and the lateral malleolus). Injections are given according to a standardized injection site scheme with horizontal and vertical spacing between the injection sites of 3 cm

Here we present our expansion and manufacturing process and report on GMP Product Quality Review data for 66 ABCB5^+^ MSC (“drug substance”) batches and 85 final drug products manufactured between January 2018 and September 2019. While process validation data have been recently published [7], we focus here on the routine GMP manufacturing process of the medicinal product, particularly on process homogeneity, comparability between batches derived from different passages and donors, and potency testing.

## Methods

### Tissue procurement and processing

Skin samples (≥ 10 cm^2^) were obtained in accordance with the German Medicines Act (“Arzneimittelgesetz”) and the German Act on Organ and Tissue Donation, Removal and Transplantation (“Transplantationsgesetz”) as discard tissues from plastic surgeries (abdominoplasties and mastopexies) from donors aged ≤ 50 years who had given written informed donor consent. Tissues from donors who tested serologically positive for HIV1/2, HBV, HCV, HTLV1/2, or syphilis were discarded.

Skin processing and stem cell production took place in an EU-GMP grade A cabinet in a grade B clean room under laminar air flow (“A in B”). Skin tissue was freed from excess subcutaneous tissue, disinfected, washed, dissected into equal pieces (about 2.5 cm^2^), and subjected to two-step enzymatic digestion using collagenase (Collagenase NB 6 GMP Grade, Nordmark, Uetersen, Germany) followed by animal component-free trypsin (Recombinant Trypsin Solution, Biological Industries, Beit Haemek, Israel). After filtration and washing/centrifugation of the filtrates, pellets were resuspended in an in-house MSC-favoring growth medium (Ham’s F-10 supplemented with fetal calf serum, l-glutamine, fibroblast growth factor-2, HEPES, hydrocortisone, insulin, glucose, and phorbol myristate acetate), pooled, and incubated in C6 cell culture plates in a cell culture incubator at 3.1% CO_2_ (as instructed by the Ham’s F-10 manufacturer, Biochrom, Berlin, Germany), 90% humidity, 37 °C.

### Assessment of cell confluency and morphology

During the whole cell expansion and subcultivation process, cell confluency and morphology were assessed visually under a phase-contrast microscope by comprehensively trained lab assistants strictly applying the four eyes principle (i.e., cross-checked by the Head of Production). Sets of standardized photographs of cultures at different stages of confluency as well as typical and untypical or artificial cell morphology are used as reference for comparison. A photograph displaying a cell monolayer at 70% confluency is shown in (Fig. S1 (see Additional file 2)).

### Cell expansion

**Fig. 1:**
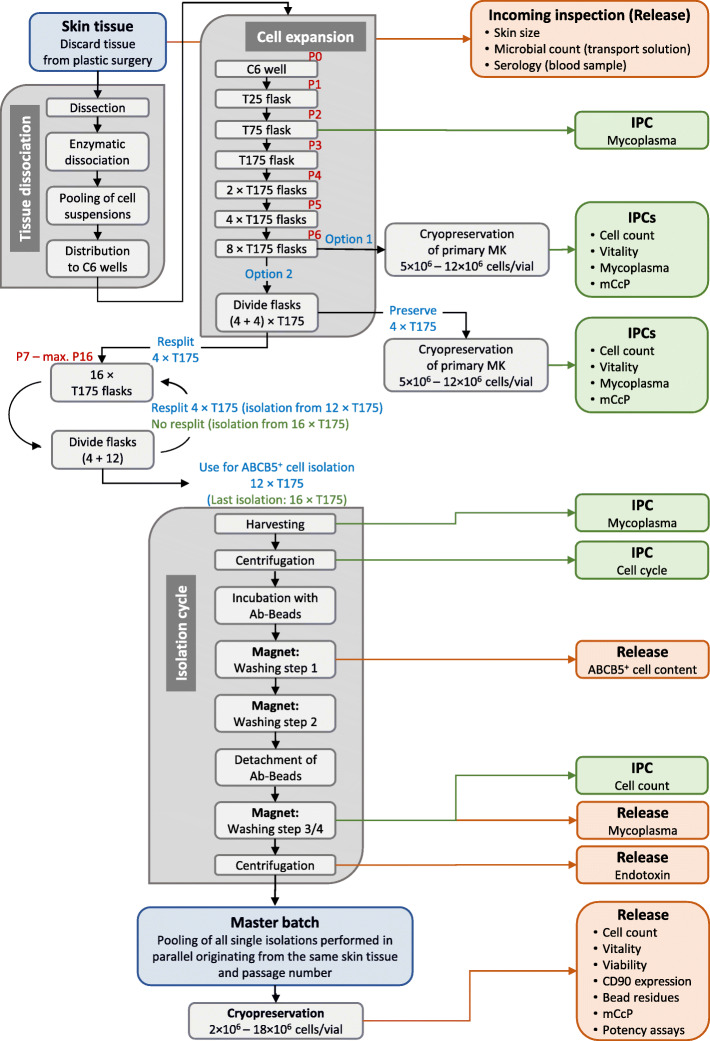
Flow chart summarizing the manufacturing process of human skin-derived ABCB5^+^ mesenchymal stem cells. *IPC* in-process control, *mCcP* microbiological control of cellular product, *MK* mixed (unsegregated) culture

Cells were expanded as unsegregated (“mixed”) cell cultures in up to 30 parallel batches from C6 well via T25 and T75 to 8 × T175 culture flasks by serial passaging (six passages). During cultivation, regular, protocol-defined medium changes facilitated depletion of non-adherent cells from the culture. Having grown to 70% confluency, cells were harvested using recombinant trypsin and transferred to the next larger culture vessel at a seeding density of 3 × 10^4^ cells/cm^2^. When 8 × T175 flasks had been reached, four flasks were harvested to be cryopreserved as primary mixed culture for internal back-up purposes. The remaining four flasks were further subcultivated to 16 × T175 flasks for magnetic separation of the ABCB5^+^ cells, respectively (Fig. 1).

### Isolation of ABCB5^+^ cells and batch pooling

After reaching 75% confluency, ABCB5^+^ MSCs were isolated using magnetic beads (micromer® TC1 Epoxy, Micromod, Rostock, Germany) coated with a mouse anti-human monoclonal antibody directed against the sequence 493–508 (RFGAYLIQAGRMTPEG) of the ABCB5 extracellular loop 3 [4] (Maine Biotechnology Services, Portland, Maine; GMP purification: Bibitec, Bielefeld, Germany; ICH Q5 [14] virus depletion and safety study: Charles River, Erkrath, Germany). The manufacturing protocol allows for multiple successive isolation cycles, by using only 12 of 16 × T175 flasks for ABCB5^+^ cell isolation, while the remaining four flasks are further subcultivated to generate 16 × T175 flasks (see Fig. 1). This cycle may be repeated until passage number 16 [7], provided that no changes in cell morphology or growth behavior occur. If required, the cryopreserved back-up mixed culture can be thawed and subcultivated for further cell production and isolation.

All ABCB5^+^ cell isolates from one and the same donor tissue that had been isolated in parallel (i.e., on the same day at the same passage number) were pooled to gain one drug substance batch. Each batch was then divided into multiple aliquots of 2 × 10^6^–18 × 10^6^ cells, which were cryopreserved (CryoStor® CS10 freeze medium, BioLife Solutions, Bothell, Washington) and stored in the vapor phase of liquid nitrogen.

### Drug product formulation

The final drug product was manufactured as an investigational medicinal product to be tested in various clinical trials (Table 1). It was formulated as a ready-to-use suspension, with total cell count and packaging size depending on the intended application. The required quantity of cryopreserved drug substance aliquots, originating from one and the donor skin tissue, were thawed, pooled, washed, and suspended in HRG solution (Ringer’s lactate solution containing 2.5% human serum albumin and 0.4% glucose) at a target concentration of 1 × 10^7^ cells/ml. The cell suspension was filled in one or multiple 1-ml polycarbonate or 10-ml polypropylene syringes (BD Plastipak™, Becton Dickinson, Heidelberg, Germany), as required, which were sealed with polyethylene closing cones (Combi-Stopper, B. Braun, Melsungen, Germany).

### In-process controls and release tests

In-process and release tests followed validated GMP-compliant procedures according to the requirements of the European Pharmacopoeia, if applicable. A schematic overview is given in Fig. 1, and the specifications are given in Table S1 (see Additional file 1).

#### Cell cycle analysis

Immediately before the magnetic separation of ABCB5^+^ cells, a sample containing about 2 × 10^6^ cells of the mixed culture were fixed with ice-cold 70% ethanol (added dropwise while vortexing) for at least 30 min at 4 °C. After washing in 0.02% EDTA, cells were resuspended in RNase A (Thermo Fisher)/propidium iodide solution and analyzed by flow cytometry (BD Accuri™ C6; Becton Dickinson, Heidelberg, Germany) using standard gating strategies.

#### Determination of ABCB5^+^ cell content

Following the ABCB5^+^ cell isolation, but before the enzymatic detachment of the microbeads (which leads to a transient loss of ABCB5 from the cell surface), ABCB5^+^ cell content was determined after incubation with an Alexa Fluor® 647-coupled donkey anti-mouse secondary antibody (Thermo Fisher, Langenselbold, Germany) targeting the anti-ABCB5 antibody used for the cell isolation. To discriminate between ABCB5^+^ cells and free bead-antibody complexes, calcein acetoxymethylester (staining metabolically active cells) was added to the cell-secondary antibody suspension before incubation. Fluorescence was measured by flow cytometry (BD Accuri™ C6, Becton Dickinson). Free, unbound bead-antibody complexes were excluded from the ABCB5^+^ cell content calculation by gating only events with high calcein fluorescence.

#### Mycoplasma testing

Supernatant and cell suspension samples were spiked with internal control DNA, and genomic DNA was isolated using the Microsart AMP Extraction Kit (Minerva Biolabs, Berlin, Germany). Isolated DNA was subjected to qPCR including positive and negative controls and 10CFU™ Sensitivity Standards for *Mycoplasma (M.) orale*, *M. fermentans*, and *M. pneumoniae* (Microsart ATMP Mycoplasma Kit; Minerva Biolabs).

#### Endotoxin testing

After the ABCB5^+^ cell isolation, microbead detachment, and washing/centrifugation of the cell suspension, a supernatant sample was diluted 1:10 with Endosafe® Limulus Amebocyte Lysate Reagent Water and transferred into an Endosafe® PTS™ cartridge (limit of detection 0.05 EU/ml), which was loaded into an Endosafe® PTS™ reader (all from Charles River, Charleston, South Carolina). The endotoxin level of the sample was calculated based on the change in optical density analyzed against an internal standard curve.

#### Determination of live cell count and vitality

Propidium iodide solution (1 mg/ml) was added to a cell suspension sample from each drug substance batch and from each produced drug product to stain dead cells. Fluorescence was measured using flow cytometry (BD Accuri™ C6) and cell count and vitality, defined as percentage of propidium iodide-excluding cells, calculated.

#### Determination of viability, CD90 expression, and bead residues

Viability, CD90 expression, and microbead residues, which might result from insufficient bead detachment or cell washing, were analyzed in parallel. From each drug substance batch, a sample was incubated with calcein acetoxymethylester and an Alexa Fluor® 647-conjugated anti-human CD90 antibody (BioLegend, London, UK). Fluorescence was analyzed by flow cytometry (BD Accuri™ C6), and the viability of the cell population (defined as percentage of metabolically active cells, i.e., cells converting calcein acetoxymethylester to calcein) and the content of CD90^+^ cells were calculated. To detect residual beads, a sample of ABCB5 antibody-conjugated beads was used to define a gate in the FSC/SSC dot plot. To exclude (false-positive) signals, only calcein-negative events in that gate were used to calculate bead residues.

#### Microbiological examination

Microbiological examination was carried out by a certified academic contract laboratory. Samples from every single cryovial of each drug substance batch and from each produced drug product were diluted in NaCl-peptone buffer, inoculated in BACT/ALERT® BPN (anaerobic) and BACT/ALERT® BPA (aerobic) culture bottles (bioMérieux, Nürtingen, Germany), and incubated in the BACT/ALERT® 3D60 (bioMérieux) microbial detection system. Positive samples were seeded onto solid culture medium immediately after detection. After 7 days of incubation, all negative samples were seeded onto solid culture medium.

#### IL-1RA secretion assay

Human monocytic cells (THP-1; LGC Standards, Wesel, Germany) were differentiated into macrophages by incubation in differentiation medium containing 150 nmol/ml phorbol 12-myristate 13-acetate for 48 h at 37 °C, 5% CO_2_. In two wells of a 24-well plate, 1 × 10^5^ macrophages were co-cultivated with 2 × 10^4^ ABCB5^+^ MSCs. In one of the two wells, M1 polarization was stimulated by adding 50 IU/ml interferon-γ (Imukin®, Clinigen Healthcare, Burton-upon-Trent, UK) at the start of cocultivation, and 50 IU/ml interferon-γ and 20 ng/ml lipopolysaccharides from *Escherichia coli* O111:B4 (Sigma-Aldrich) after 24 h. After 48 h, supernatants were collected and analyzed for IL-1RA using a colorimetric sandwich ELISA kit (Quantikine®, R&D Systems, Abingdon, UK) according to the manufacturer’s instructions.

#### VEGF secretion assay

ABCB5^+^ MSCs (3 × 10^5^) were seeded into a culture dish in stem cell medium and incubated under hypoxic conditions (1% O_2_) at 37 °C. After 48 h, supernatants were collected and analyzed using the Invitrogen VEGF Human ELISA Kit (Thermo Fisher) according to the manufacturer’s instructions.

#### Tube formation assay

ABCB5^+^ MSCs (1 × 10^5^ and 1.5 × 10^5^) were seeded in two wells of a 24-well plate coated with Geltrex® basement membrane matrix (Thermo Fisher) and incubated in stem cell medium for 19–22 h at 37 °C, 3.1% CO_2_. Tube structures were photographed using an inverted microscope (40× final magnification; DM IL LED, Leica, Wetzlar, Germany) equipped with a digital camera (DFC320, Leica) and semi-quantitatively classified into six (A–F) categories, ranging from A = tubular branches of several cells forming a defined network-like structure to F = no tubular branches visible (Fig. S2 (see Additional file 2)), with A–C being considered as successful angiogenic differentiation. For assay validation, human umbilical vein endothelial cells (Thermo Fisher) and human skin melanoma cells (SK-MEL-28, ATCC® HTB-72™, LGC Standards, Wesel, Germany) were used as positive and negative controls, respectively.

### Determination of MSC markers

In addition to routine determination of CD90^+^ cell content (see the “In-process controls and release tests” section), the batches intended for use at study sites in the Czech Republic in the multinational clinical trial on peripheral artery disease (NCT03339973, Table 1) were additionally tested for expression of CD73, CD105, und CD44 [5, 15], as required by the Czech national drug authority (State Institute for Drug Control, SÚKL). To this end, cell suspension samples were incubated with a fluorescein isothiocyanate (FITC)-conjugated anti-human CD73 antibody, an Alexa Fluor® 647-conjugated anti-human CD105 antibody, and a FITC-conjugated anti-human CD44 antibody (all from BioLegend), respectively. Fluorescence was measured by flow cytometry (BD Accuri™ C6) and percentages of CD73^+^ CD105^+^ and CD44^+^ cells were calculated. Pre-specified acceptance criteria were CD73, ≥ 95% [5]; CD105, determined and declared; and CD44, determined and declared.

## Results

### Starting material

Between July 2017 and September 2019, we received skin donations from twelve donors obtained in two dermatological clinics approved as tissue retrieval facilities according to §20b German Medicines Act (“Arzneimittelgesetz”). Ten of these skin donations were left-over tissues from abdominoplasties, the remaining two from plastic breast surgeries. All donors were female and between 28 and 49 years of age.

Five of the twelve skin specimens were rejected and discarded before or retroactively during processing for various reasons including fungal contamination of the transport medium (*n* = 2), bacterial contamination of the cleanroom (*n* = 1), and cell morphology changes during culture (*n* = 2). All data in this report relate to the remaining seven skin donations that fulfilled the specifications for tissue release.

### Cultivation time

**Fig. 2:**
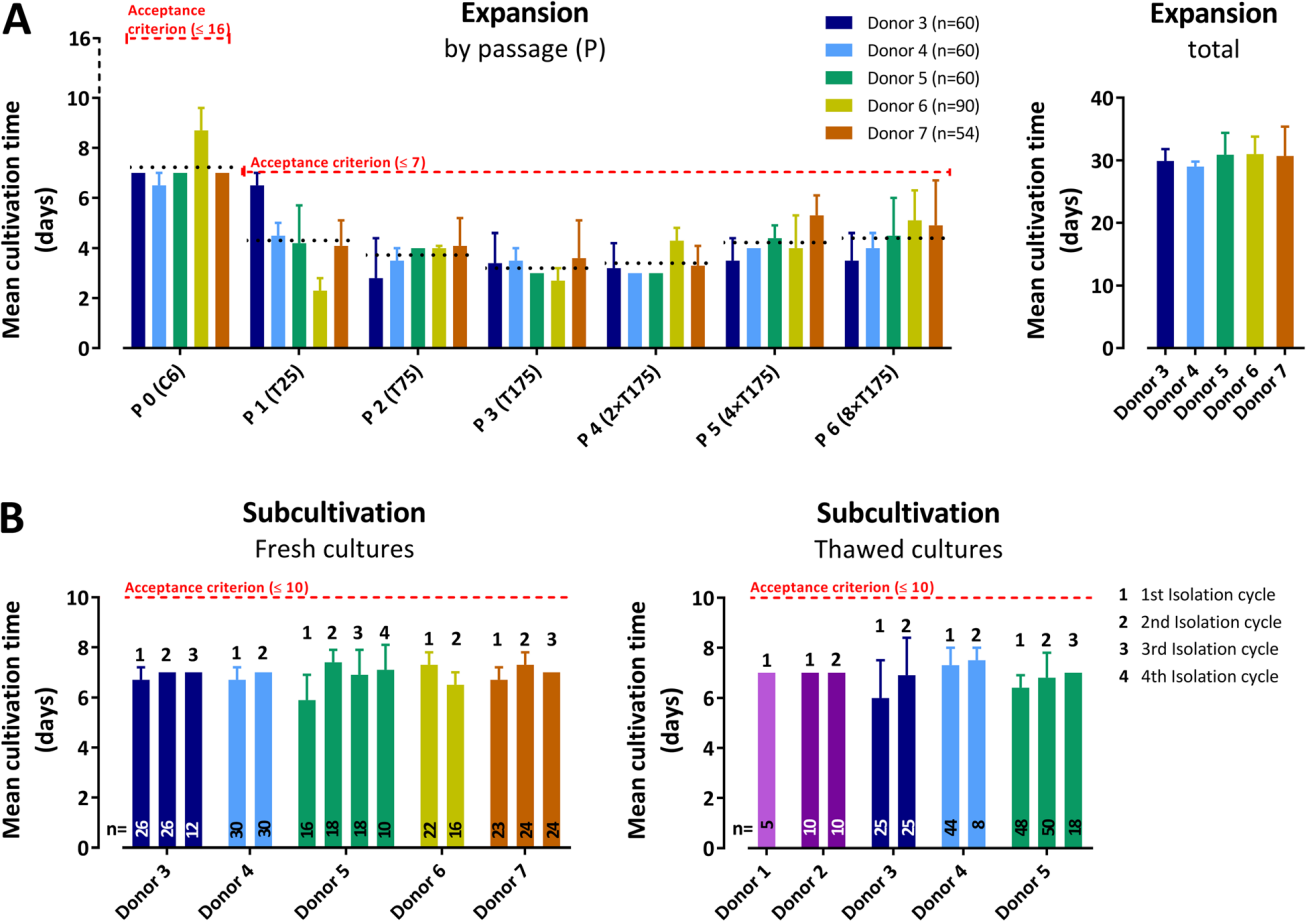
Intra-donor mean (+ SD) cultivation times of mixed (unsegregated) cell cultures. **a** Mean cultivation times during cell expansion from culture initiation until passage 6 shown by passage (left panel) and in total (right panel). Black dotted lines indicate passage means. **b** Mean duration of subcultivation (from 4 to 16 × T175 culture flasks) before isolation of the ABCB5^+^ cells from fresh (left panel) and from previously cryopreserved/thawed cultures (right panel) for up to four isolation cycles. Fresh cultures: isolation cycles 1–4 correspond to passages 7–10; thawed cultures: isolations cycles 1–3 correspond to passages 9–11. Data represent all expansions and isolation cycles performed between January 2018 and September 2019; therefore, for donors 1 and 2, only thawed cultures are shown (because expansion and isolation from fresh cultures had been performed before January 2018). Red dashed lines indicate the upper time limit; if a culture had not reached target confluency within this prespecified period, it would have been discarded

Homogeneity of the expansion process was evaluated by analysis of the cultivation time, i.e., the time periods between passaging and reaching ≥ 70% confluency (as the threshold for the next split/passaging of the culture) in the culture vessel. Figure 2a summarizes the cultivation times until passage 6 for 324 expansions carried out between January 2018 and September 2019. All cultures met the specified upper time limit (16 days for cultivation until the first passage and 7 days for the subsequent passages; based on validation data showing that cultures that require longer time periods to reach the target confluency will nearly totally stop growth at later stages of the expansion process due to senescence, differentiation and morphology changes). The mean cultivation time from initiation of culture until generation of eight confluent T175 flasks was 30.5 days, with the inter-donor range of 29.0 to 32.4, corresponding to a maximal difference of means between donors of 10.2% (right panel).

Next, the time intervals needed for subcultivation of 4 × T175 flasks to 16 × T175 flasks at ≥ 75% confluency (as the threshold for isolation of the ABCB5^+^ cells from the unsegregated subcultures) were evaluated. Figure 2b summarizes the results for immediately subcultivated (“fresh”) cultures (left panel) and for primary cultures that had been cryopreserved (“thawed”) (right panel). Similar to what was seen during the expansion process, the specified upper limit (10 days) for subcultivation was met in all cases. Also, consistently low intra-donor standard deviations and narrow inter-donor ranges (fresh cultures, 5.7–7.4 days; thawed cultures, 6.4–7.5 days) indicate a high level of homogeneity of the subcultivation process within as well as between the different donors, irrespective of whether fresh cultures or cultures that had been cryopreserved and thawed prior to subcultivation were used.

### Cell cycle phase distribution

**Fig. 3:**
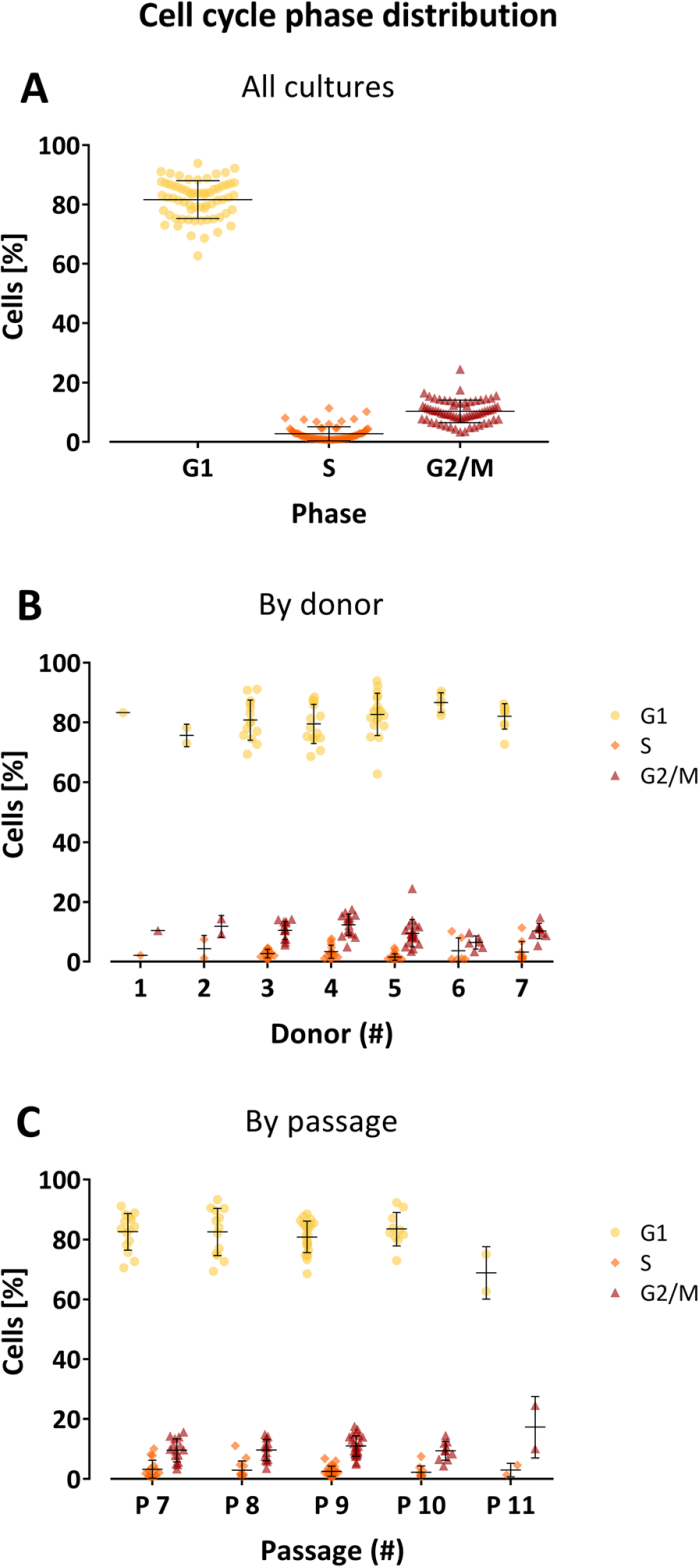
Cell cycle analysis of the primary cultures used for production of 66 drug substance batches. **a** Percentages of cells in G1, S, and G2/M phases shown for each culture. **b** Data from (**a**) grouped by donors. **c** Data from (**a**) grouped by passage number. Error bars indicate means ± SD

Cell cycle analysis, which is performed as an in-process control before isolation of the ABCB5^+^ cells from the mixed culture, revealed a phase distribution of 82% (± 6%) of cells in G1, 3% (± 2%) in S, and 10% (± 4%) in G2/M phase (Fig. 3a). The distribution pattern did not differ between donors (Fig. 3b) nor change during passaging (Fig. 3c).

### Drug substance analyses

Between January 2018 and September 2019, 728 ABCB5^+^ cell isolates were obtained, which were pooled to 66 “drug substance” batches. Of these, 47 batches, made up of 548 single cell isolates in total, were released for final drug production.

#### ABCB5^+^ cell isolate evaluation

The mean live cell count of the ABCB5^+^ cell isolates varied between donors, ranging from 19.0 × 10^6^ ± 5.5 × 10^6^ (donor 2) to 48.6 × 10^6^ ± 16.9 × 10^6^ (donor 7). These inter-donor variations in ABCB5^+^ MSC yield did not correlate with donor age (Table S2 (see Additional file 1)).

**Fig. 4:**
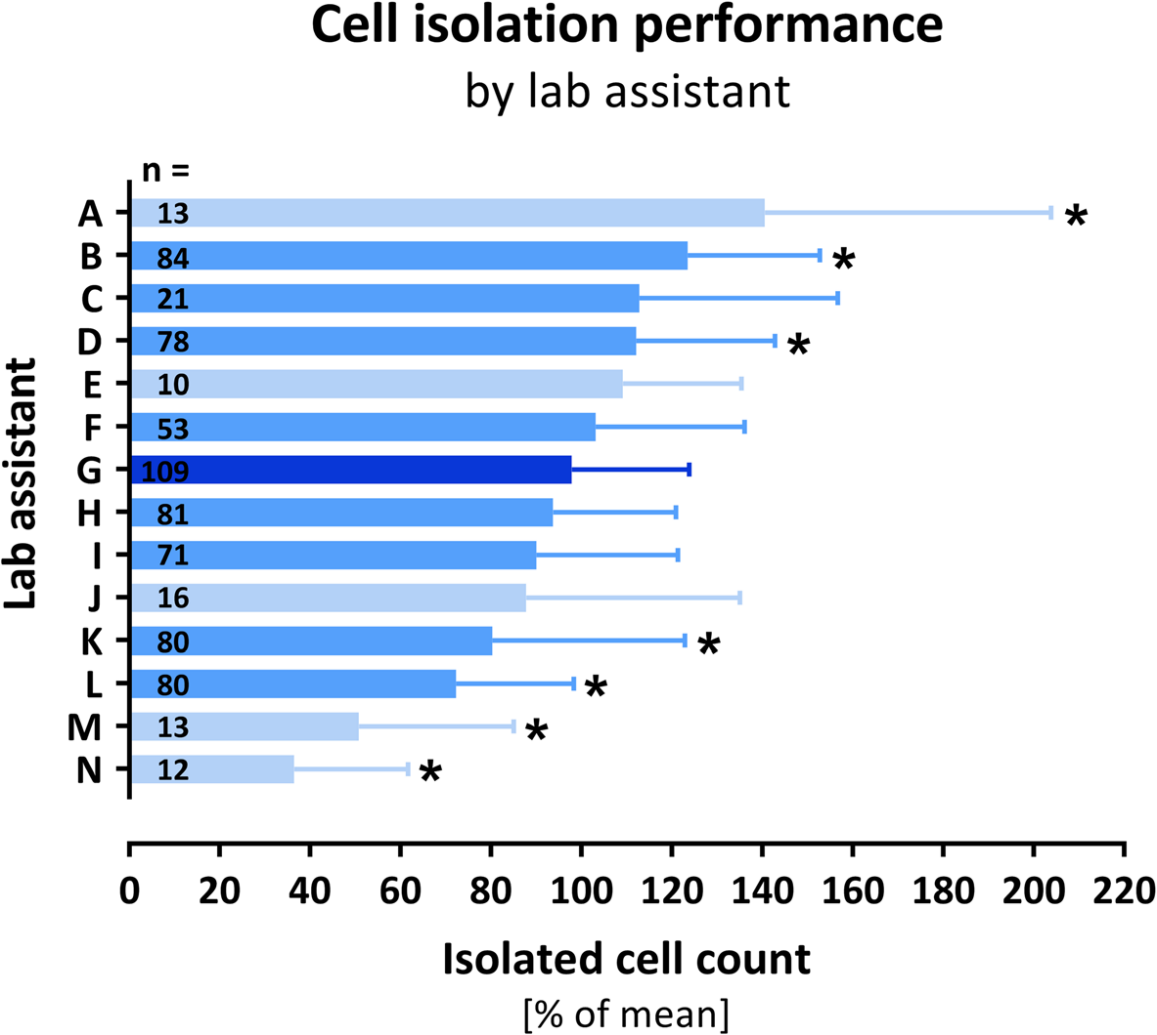
Lab-assistant-specific performance of ABCB5^+^ cell isolation. Performance is expressed as the mean percentage (± SD) from the mean live cell count of all ABCB5^+^ cell isolates that were afterwards pooled to the same drug substance batch. Cell counting was performed strictly independently from the lab assistant by the quality control department using a standardized automated method (flow cytometry). Included in this analysis are all lab assistants who had carried out at least 10 isolations (*n* = 721 isolations in total), with the less experienced lab assistants (10–19 isolations) colored in light blue and the more experienced (≥ 20 isolations) in median blue. *p < 0.05 (one-way ANOVA with Bonferroni’s post hoc test) vs. the most experienced assistant (G, dark blue). Note that the letters A–N identifying the lab assistants are arbitrarily assigned based on the order of data arrangement and do not match with those used in Fig. 6

In order to identify potential lab assistant-related influences on the ABCB5^+^ cell isolation performance, we aimed to assess to what extent the cell count isolated by the assistant differs from the mean cell count of all batches that were isolated in parallel (i.e., on the same day from the same donor and same passage) for each lab assistant. As shown in Fig. 4, the amount of isolated ABCB5^+^ cells by each lab assistant expressed as the mean percentage from the mean live cell count of all isolates that were afterwards pooled to the same drug substance batch varied between the lab assistants from about 40 to 140% (for all assistants who had carried out at least 10 isolations). When only the more experienced lab assistants (i.e., who had carried out at least 20 isolations) were considered, the range was 75–125%.

To limit a potential impact of variability between the ABCB5^+^ cell isolates on drug substance batch homogeneity and quality, each set of parallelly obtained isolates is only released as a whole for drug substance batch pooling, if the coefficient of variation of the cell count does not exceed 50%; otherwise, only those isolates of the set are released, whose cell counts do not differ by more than 50% from the mean cell count. Of 66 cell isolate sets that were pooled to 66 drug substance batches, 64 sets proved entirely “poolable” and were released as a whole. From the remaining 2/66 cell isolate sets, 2/10 and 3/6 isolates, respectively, had to be rejected due to > 50% deviation from the mean cell count of the set, so that 8/10 and 3/6 isolates, respectively, could be released for drugs substance batch pooling.

#### Drug substance batch evaluation

Of the 66 drug substance batches that were produced between January 2018 and September 2019 (Table S3 (see Additional file 1)), 9 batches were rejected by the quality control unit due to out-of-specification results [limit value (< 1 CFU) exceedance for environmental microbiological monitoring (*n* = 1), release parameter deviation from the specified acceptance criterion (*n* = 8; Table 2)], 10 batches were used for research and development purposes, and 47 batches were released for final drug product manufacturing.

**Table 2:** Results from drug substance release testing (*n* = 66 batches derived from 7 donors)

Parameter	Specification	Result	Deviations	
			absolute	%
ABCB5^+^ cell content	≥ 90%	97.72 ± 1.88%	1^a^	1.5^a^
Mycoplasma	Not detectable (< 10 CFU/ml)	Not detectable in 65/66 batches	1	1.5
Endotoxin level	≤ 2 EU/ml	≤ 2 EU/ml in 66/66 batches	0	0
Live cell count	n.a.^b^	275.33 × 10^6^	n.a.^b^	n.a.^b^
Deviation of live cell count from expected value^c^	≤ 30%	≤ 30% in 46/49 batches^d^	3	6%^d^
Cell vitality^e^	≥ 90%	98.60 ± 0.62%	0	0
Cell viability^f^	≥ 90%	99.45 ± 0.64%	0	0
CD90^+^ cell content	≥ 90%	99.45 ± 0.50%	0	0
Bead residues	≤ 0.5%	0.04 ± 0.05%	0	0
Microbiological control	No growth	No growth in 66/66 batches	0	0
IL-1RA secretion	> 125 pg/ml and ratio_stim/unstim_ > 1	> 125 pg/ml and ratio_stim/unstim_ > 1 in 66/66 batches	0	0
VEGF secretion	> 46.9 pg/ml	> 46.9 pg/ml in 66/66 batches	0	0
Angiogenic differentiation	Capillary structures in one or both seeded cell concentrations	positive in 63/66 batches	3	4.5%

Numeric results are given as mean ± SD

*n.a.* not applicable

^a^For one batch, measurement procedure was not fully GMP-conform. Although this did not affect the result, the batch was not released for drug production

^b^Acceptance criteria for cell count are only defined for the aliquots into which the drug substance batches are divided for cryostorage, not for the batch as a whole

^c^Expected value = sum of the live cell counts of all ABCB5^+^ cell isolates that were pooled to produce the drug substance batch

^d^The criterion “Deviation of live cell count from expected value” was implemented only from donor 4 on, which is the reason for the lower sample number (*n* = 49)

^e^Vitality was defined as percentage of live cells, defined as propidium iodide-excluding cells

^f^Viability was defined as percentage of metabolically active cells, defined as cells converting calcein acetoxymethylester to calcein

**Fig. 5:**
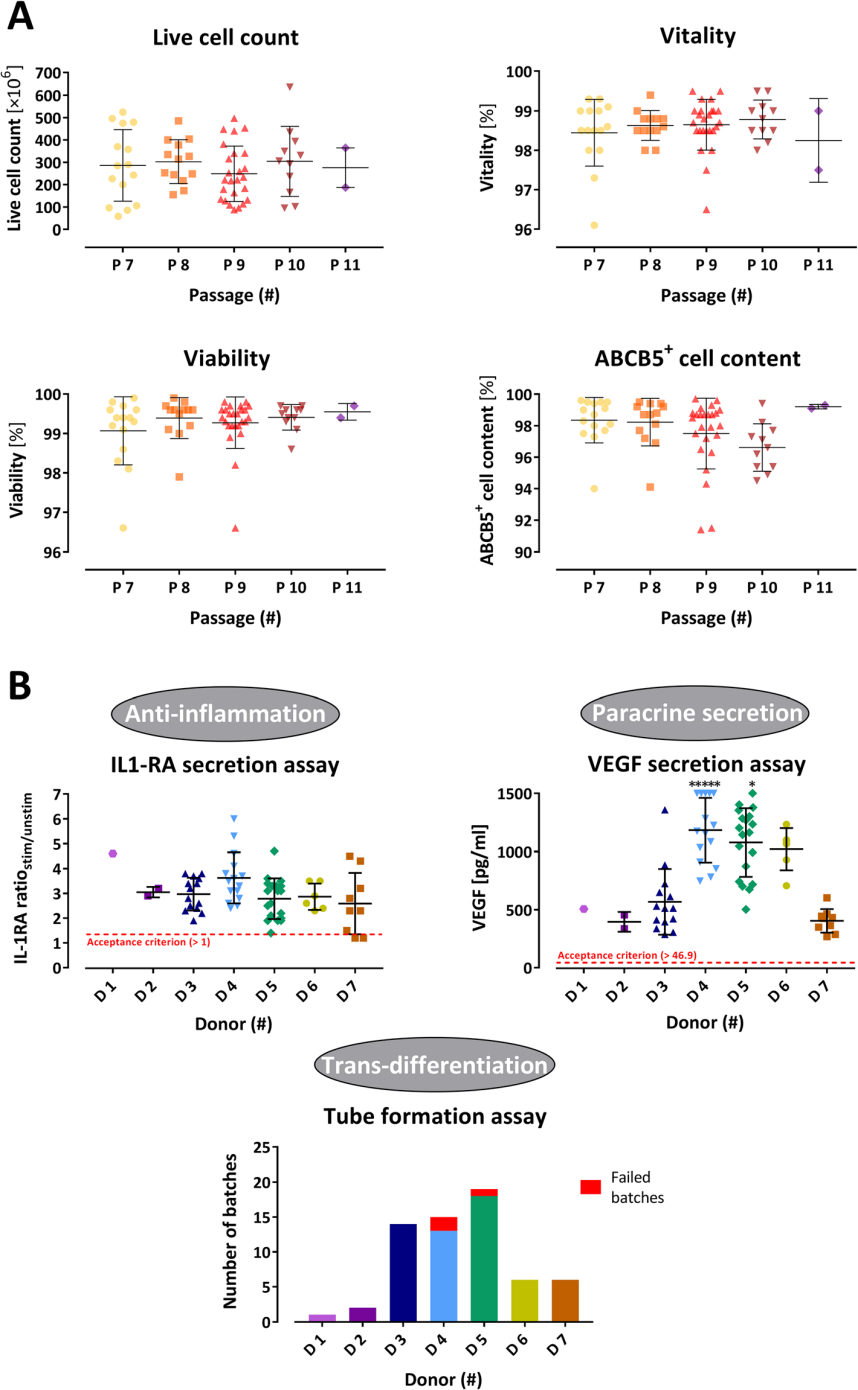
Results from drug substance batch quality control analyses. **a** Live cell count, vitality, viability and ABCB5^+^ cell content of the 66 produced drug substance batches as determined before cryopreservation, shown by the number of the passage at which the ABCB5^+^ cells were isolated from the mixed culture. **b** Anti-inflammatory and proangiogenic potency of ABCB5^+^ mesenchymal stromal cells (MSCs) as measured by secretion of interleukin-1 receptor antagonist (IL-1RA) after stimulation by M1-polarized macrophages (IL-1RA secretion assay; given as ratio to IL-1RA secretion observed with unstimulated ABCB5^+^ MSCs), secretion of vascular endothelial growth factor (VEGF) after 48-h culture under hypoxic conditions (VEGF secretion assay; *value above upper limit of assay range, i.e., > 1500 pg/ml), and capillary structure formation in extracellular matrix (tube formation assay; red bar segments indicate the batches that failed to clearly form capillary structures). Error bars indicate means ± SD

To detect a potential impact of the level of cell passaging on drug substance batch homogeneity and quality, the release parameters live cell count, vitality, viability, and ABCB5^+^ cell content were also evaluated in relation to the passage number at which the ABCB5^+^ cells were isolated from the mixed culture. Neither of these parameters showed any passage number-dependent variation (Fig. 5a).

#### Potency testing

All 66 drug substance batches fulfilled the specification for anti-inflammatory (IL-1RA secretion in response to stimulation by M1-polarized macrophages) and pro-angiogenic [VEGF secretion after 48 h hypoxia potency (Table 2)], with donor means ranging from 2.6 ± 2.0 (donor 7) to 4.6 (*n* = 1) (donor 1) for IL-1RA ratio_stim/unstim_ and from 396 ± 85 pg/ml (donor 2) to 1183 ± 278 pg/ml (donor 4) for VEGF supernatant concentration (Fig. 5b). All but 3 (4.5%) of the 66 drug substance batches tested positive in the endothelial trans-differentiation assay as shown by capillary structure formation by at least one of two cell concentrations seeded in extracellular matrix (Fig. 5b, Table 2).

#### MSC marker expression

Determination of MSC marker expression in 17 drug substance batches derived from 3 donors (i.e., the batches intended for use at study sites in the Czech Republic, as required by the Czech national drug authority) revealed that the markers CD90, CD73, CD105, and CD44 were expressed by 99.6 ± 0.9%, 99.8 ± 0.2%, 99.5 ± 0.8%, and 99.5 ± 0.9% of cells, respectively. All batches met the pre-specified criteria for batch release.

### Final drug product

Between January 2018 and September 2019, 85 drug products used as investigational medicinal products in clinical trials were manufactured (for overview see Table 1). All products met the pre-specified criteria (Table S1) for batch release.

**Fig. 6:**
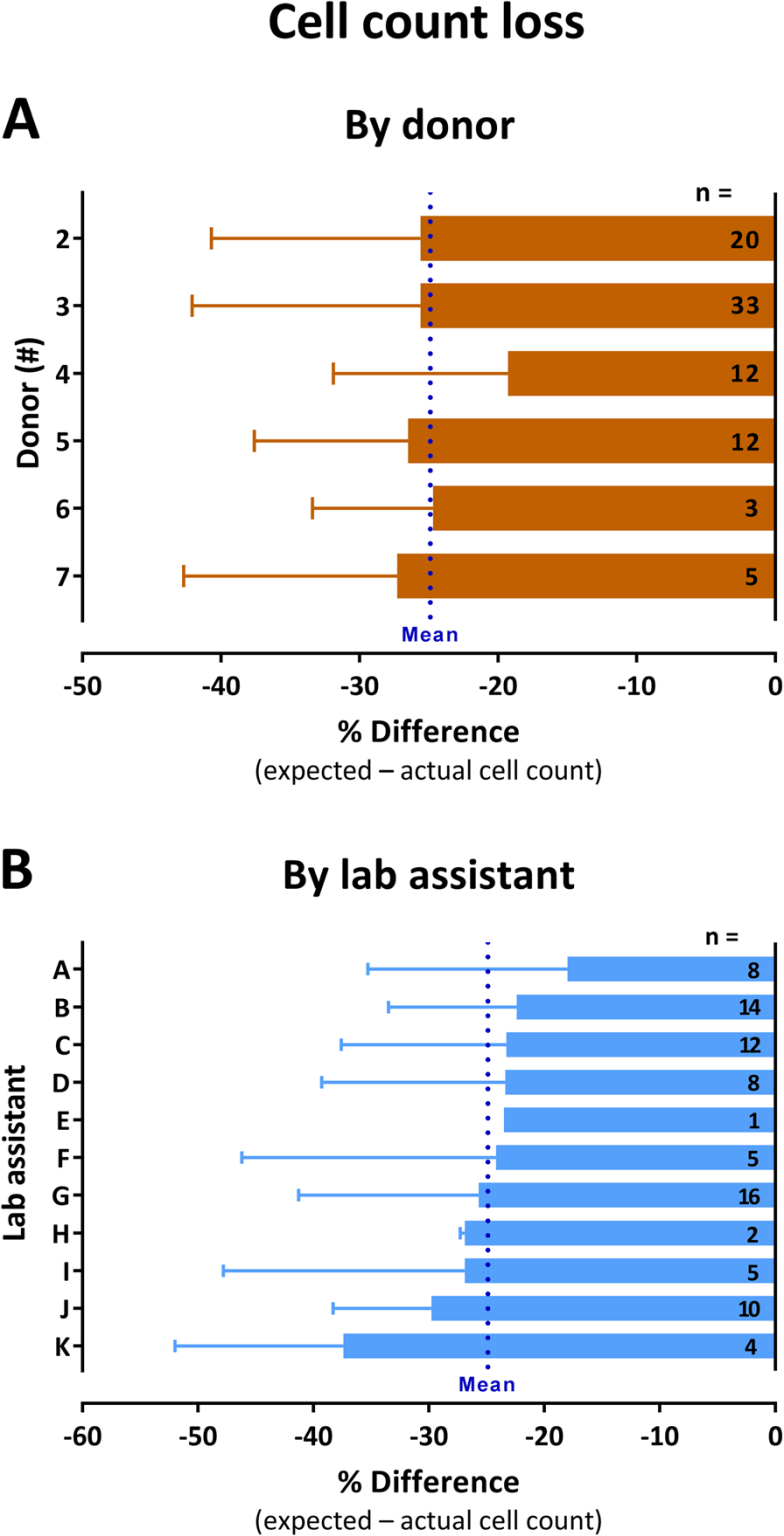
Cell count loss during final drug product manufacturing from cryopreserved drug substance aliquots. **a** Cell count loss shown by tissue donor. **b** Cell count loss shown by lab assistant. Cell count loss is expressed as percentage difference between the expected (as calculated from the number and the cell content of the aliquots) and the actual final cell count. Shown are means ± SD of n manufactured products; blue dotted lines indicate mean cell count loss of all 85 produced drug products. Note that the letters A–K identifying the lab assistants are arbitrarily assigned based on the order of data arrangement and do not match with those used in Fig. 3

Final product manufacturing process was associated with a mean cell count loss (most likely related to freeze/thaw losses and process steps such as transfer, washing, and centrifugation) of 24.9 ± 14.5%, which was comparable between the different skin donors, with donor means ranging from 19.3 ± 12.6% (*n* = 12, donor 4) to 27.3 ± 15.4% (*n* = 5, donor 7) (Fig. 6a). Cell count loss was also comparable between 10 out of the 11 lab assistants who carried out the formulation processes including all the critical process steps at which process-related cell loss might occur, with assistant-related mean cell count loss ranging from 18.0 ± 17.3% (*n* = 8) to 29.8 ± 8.5% (*n* = 10). Only for one lab assistant, a greater mean cell loss (37.4 ± 14.6%, *n* = 4) as compared to the other assistants was observed (Fig. 6b).

Determination of MSC marker expression in 5 drug products derived from 3 donors (i.e., the drug products intended for use at study sites in the Czech Republic, as required by the Czech national drug authority) revealed CD90, CD73, CD105, and CD44 expression by 99.9 ± 0.1%, 100 ± 0.1%, 99.5 ± 0.5%, and 100 ± 0.0% of cells, respectively.

## Discussion

Aiming at enabling safe, effective, and reproducible therapeutic use of donor-derived dermal ABCB5^+^ MSCs, we developed and validated a GMP-compliant ex vivo expansion and manufacturing process to generate an ATMP derived from skin tissue donations that guarantees reliable and reproducible cell quality and functionality while being deliverable as an instantly available off-the-shelf product.

From experience with MSCs derived from bone marrow or adipose tissue, it is known that age and morbidities of the donor can impair the number, viability, proliferation capacity, maximum life span, and functional properties such as differentiation potential and proangiogenic factor secretion [16–24]. For dermal ABCB5^+^ MSCs, an age-dependent decrease in their number in situ from 3.2% of total dermal cells in the skin of young individuals to 1.6% of total dermal cells in the skin of individuals > 70 years has been observed [12]. Together, these observations prompted us to limit donor age to ≤ 50 years and to define strict donor health requirements. Nevertheless, even MSCs derived exclusively from young and healthy donors have been described to exhibit donor-dependent variations in molecular status, growth behavior, and functional characteristics [25, 26], which, ultimately, could result in donor-related batch-to-batch variability. Other factors that could potentially contribute to unwanted product inhomogeneity may be related to variations in the expansion and manufacturing process such as passage number or lab assistant experience. In this context of a situation where the “process is the product” [1, 27], it is crucial to closely monitor the entire process at each step from the starting material until the final drug product to detect and eliminate any factors that would impair the homogeneity of the cell therapy product.

During cultivation, potential donor and/or passage number-related variations in the proliferative capacity of the cultured cells were monitored by assessing the time needed from passaging to reach the target confluency for each passage. In all cases, the specified upper time limits during both expansion (passages 1–6) and subcultivation (up to passage 11, depending on the number of isolation cycles performed) were met (Fig. 2). Low intra-donor standard deviations and generally narrow inter-donor ranges indicate high homogeneity of the proliferative behavior of the cells within and between the different donors, respectively. This is supported by previous cell division data collected during the process validation phase, which revealed a constant mean division rate of 0.35 divisions per day across 15 donors and even up to 16 passages [7]. It should be noted that the number of isolation cycles (which accounted for up to 11 passages at maximum) for each donor depended on the company’s production plan and were, in most cases, not restrained by cell culture-related phenomena such as changes in cell morphology or growing behavior.

Low variation within donors and a high degree of comparability between donors was also seen for the cell cycle distribution of ABCB5^+^ MSCs (Fig. 3). The phase distribution pattern (82 ± 6%, 3 ± 2%, and 10 ± 4% for G1, S, and G2/M phase, respectively) resembled extensive data collected during process validation (503 analyses from 260 donors: 72.4 ± 8.5%, 4 ± 2.9%, and 15 ± 8.5% for G1, S, and G2/M phase, respectively) [7] and did not change during passaging until passage 11 (Fig. 3c). Taken together with previous RNA sequence analysis data revealing that the expression of various stemness and mesenchymal marker genes was not altered in ABCB5^+^ MSCs expanded to high passage numbers (> 10) as compared to low passage numbers (2–3) [6] and microarray data (GEO accession No. GSE145589) showing that the gene expression profile of ABCB5^+^ MSCs did not significantly change over five delta passages up to passage 16 [7], these results support that proliferation capacity and MSC properties of ABCB5^+^ MSCs are maintained during ex vivo expansion up to comparatively late passages.

The isolation process of ABCB5^+^ MSCs from the expanded mixed cell culture was evaluated by assessing the homogeneity of the ABCB5^+^ cell isolates and the quality of the drug substance batches produced thereof. Obviously, the cell isolates varied considerably in their mean live cell count between the different donors, with the highest donor mean (48.6 × 10^6^ ± 16.9 × 10^6^ cells per isolate) amounting to more than twofold of the lowest donor mean (19.0 × 10^6^ ± 5.5 × 10^6^ cells per isolate) (Table S2). This donor-related variation was neither dependent on donor age, sex (all donors were female) nor skin tissue localization (abdomen vs. mamma) (Table S2) and may therefore reflect other, likely individual, differences between donors. In this regard, please note that the use of female donors in this study occurred by chance (the plastic surgeries in our collaborating tissue retrieval facilities from which we obtained our starting materials were coincidentally underwent by female subjects) and does not reflect a preference of female to male donors. Variations in the mean live cell count of the cell isolates were also observed between the different lab assistants who performed the ABCB5^+^ cell isolation. From our experience, a certain degree of variation can be expected based on the various processing steps such as harvest, isolation, washing, resuspension, and sampling. As it turned out, the degree of variation declined with increasing experience of the lab assistant. In contrast to lab assistant-related variability, the number of passages that a culture had undergone until ABCB5^+^ cell isolation did not appear to have impact on the live cell count of the isolate, since vitality and viability of the isolated cells did not change with increasing passage numbers (Fig. 5a).

While lab assistant-related variability in the live cell counts between the cell isolates could likely be decreased by enhancement of lab staff training, potential donor-individual influences cannot be eliminated prospectively. Therefore, to limit a potential impact of ABCB5^+^ cell isolate variability on the homogeneity of the drug substance batches produced thereof, we defined that the coefficient of variation of the cell count of a set of ABCB5^+^ cell isolates may not be greater than 50% for it to be pooled into one drug substance batch (otherwise only the isolates that did not vary by more than 50% from the mean cell count of the whole set of isolates intended to be pooled were ultimately pooled into a drug substance batch). Applying this criterion, 64 of 66 isolate sets could be pooled as a whole, the remaining two isolate sets at least in part. Thus, in the future, this criterion could be defined more strictly in order to further increase process homogeneity. Of note, drug substance batch quality was not negatively affected by this variability. Specifically, only 8 out of 66 drug substance batches were rejected due to specification failure (Table 2).

A key component in the quality assessment of cellular therapy products is the evaluation of potency, in order to guarantee biological functionality that is predictive of clinical effectiveness. Considering that MSCs exert their therapeutic effects through a variety of pathways that are induced upon interaction with the host microenvironment, which has constituted the paradigm of MSCs to function as site-regulated, patient-specific “injury drugstores” [28], evaluation of a single pathway is thought insufficient to predict clinical effectiveness [2]. We therefore developed and implemented three potency assays reflecting the three clinically most relevant biologic modes of action of ABCB5^+^ MSCs: (i) IL-1RA secretion assay after co-culture with M1-polarized macrophages to predict the anti-inflammatory potency in M1 macrophage dominated inflammatory tissue environment, (ii) VEGF secretion assay in hypoxic culture conditions to estimate the pro-angiogenic bioactivity in ischemic tissue environment, and (iii) tube formation assay to demonstrate the endothelial differentiation and blood vessel-forming capacity of the produced cells. Except for 3 batches that failed to clearly form capillary structures in the tube formation assay, all drug substance batches fulfilled the specified criteria for all three assays (Fig. 5b, Table 2). Notably, however, while these assays were validated to reliably detect biological activity and thus qualitatively confirm the potency of ex vivo-expanded ABCB5^+^ MSCs, a quantitative correlation between the assay values and the strength of clinical efficacy remains to be elucidated. Comparisons between the outcomes of the ongoing clinical trials and the assay data results will offer valuable clues on quantitative potency prediction.

## Conclusions

Taken together, the present GMP Product Quality Review data demonstrate the high robustness of our cell expansion and manufacturing process, enabling the generation of a homogenous, standardized, highly pure (97.7% ABCB5^+^ MSCs), off-the-shelf available ATMP with proven potency to be used for therapy of a variety of skin-related and other, including systemic, inflammatory and/or degenerative conditions. At present, clinical trials to confirm the preclinical safety and efficacy data in various clinical indications are underway. Beyond their clinical informative value, the outcomes of these trials will also be used to refine the manufacturing process design, aiming at continuously improving and optimizing the procedures and control measures and, consequently, the product.

## Supplementary information

**Additional file 1 : Table S1.** Tests and specifications for drug substance batch and final drug product release. **Table S2.** Mean live cell count per ABCB5^+^ cell isolate by donor. **Table S3** Results from drug substance batch release testing by donor.

**Additional file 2 : Fig. S1.** Photographic documentation of the cell monolayer at 70% confluency. Original total magnification × 200. **Fig. S2.** Tube formation assay on extracellular matrix gel. ABCB5^+^ mesenchymal stem cells (1 × 10^5^ and 1.5 × 10^5^) were seeded in two wells of a Geltrex™-coated 24-well plate and incubated for 19–22 h. Tube formation was evaluated visually according to the following categories: **A** tubular branches of several cells forming a defined network-like structure; **B** tubular branches of several cells clustering together forming broad strands, formation of syncytia, areas of high cellular density lacking formation of tubular branches; **C** cells clustering together, building nodes and forming tubular branches that connect the nodes with each other; **D** only sporadic cells form tubular branches, partial node formation, but no or nearly no connections between nodes, no or only sporadic apoptotic cells; **E** largely apoptotic cells, no or only sporadic tubular branches; **F** no tubular branches. Human umbilical vein endothelial cells (HUVEC) and human skin melanoma cells (SK-MEL-28) served as positive and negative controls, respectively. Categories A–C are considered as successful angiogenic differentiation. Original total magnification × 40.

## Data Availability

The datasets generated and/or analyzed during the current study are available from the corresponding author on reasonable request.

## Abbreviations

ABCB5: ATP Binding Cassette Transporter, Subfamily B, Member 5
ATMP: Advanced-therapy medicinal product
GMP: Good Manufacturing Practice
HRG: Ringer’s lactate solution containing human serum albumin and glucose
IL-1RA: Interleukin-1 receptor antagonist
MSC: Mesenchymal stromal cell
VEGF: Vascular endothelial growth factor
