# Supplementary material for: Process data of allogeneic ex vivo-expanded ABCB5+ mesenchymal stromal cells for human use: off-the-shelf GMP-manufactured donor-independent ATMP
Source: Stem Cell Res Ther. 2020 Nov 16;11:482. doi: 10.1186/s13287-020-01987-y (PMC7667860; doi:10.1186/s13287-020-01987-y)
Supplement: Supplementary file 1 — Additional file 1 : Table S1. Tests and specifications for drug substance batch and final drug product release. Table S2. Mean live cell count per ABCB5+ cell isolate by donor. Table S3 Results from drug substance batch release testing by donor. [file 13287_2020_1987_MOESM1_ESM.docx]

**Table S1** Tests and specifications for drug substance batch and final drug product release

| **Parameter** | **Method** | **Specification** | |
| --- | --- | --- | --- |
|  |  | **Drug substance** (isolated ABCB5^+^ MSCs) | **Final drug product** (ABCB5^+^ MSCs in HRG, 1×10^7^ cells/ml) |
| ABCB5^+^ cell content | Flow cytometry | ≥ 90% | ≥ 90%^a)^ |
| Mycoplasma | Nucleic acid amplification test (2.6.7/2.6.21 Ph. Eur.) | not detectable (< 10 CFU/ml) | not detectable (< 10 CFU/ml)^a)^ |
| Endotoxin level | Limulus amebocyte lysate test (2.6.14 Ph. Eur.) | ≤ 2 EU/ml | ≤ 2 EU/ml^a)^ |
| Live cell count | Flow cytometry (2.7.29/2.7.24 Ph. Eur.) | 2×10^6^–18×10^6^/cryovial | variable (depending on intended application) |
| Deviation of live cell count from expected value^b)^ | Calculation | ≤ 30% | n.a. |
| Cell vitality | Flow cytometry (2.7.29/2.7.24 Ph. Eur.) | ≥ 90% | ≥ 75% |
| Cell viability | Flow cytometry (2.7.29 Ph. Eur.) | ≥ 90% | ≥ 90%^a)^ |
| CD90^+^ cell content | Flow cytometry | ≥ 90% | ≥ 90%^a)^ |
| Bead residues | Flow cytometry | ≤ 0.5% | ≤ 0.5%^a)^ |
| Microbiological control | BacT/ALERT® (adapted to 2.6.27 Ph. Eur.) | no growth | no growth |
| IL‑1RA secretion | ELISA | > 125 pg/ml and Ratio_stim/unstim_ >1 | > 125 pg/ml and Ratio_stim/unstim_ >1^a)^ |
| VEGF secretion | ELISA | > 46.9 pg/ml | > 46.9 pg/ml^a)^ |
| Angiogenic differentiation | Tube formation assay | Capillary structures in one or both seeded cell concentrations | Capillary structures in one or both seeded cell concentrations^a)^ |

*MSC* mesenchymal stem cells, *HRG* Ringer’s lactate solution containing human serum albumin and glucose, *n.a.* not applicable, *Ph. Eur.* European Pharmacopoeia

^a^Parameter is not tested in the final drug product; value is adopted from drug substance release testing. Transferability of the result from drug substance release testing onto the final drug product was demonstrated in stability studies and method validations

^b^Expected value = sum of the live cell counts of ABCB5^+^ cell isolates that were pooled to produce the drug substance batch

**Table S2** Mean live cell count per ABCB5^+^ cell isolate by donor

| **Donor** | | | | **ABCB5^+^ cell isolates** | |
| --- | --- | --- | --- | --- | --- |
| **#** | **Sex** | **Age** (years) | **Skin tissue origin** | **Total number of ABCB5^+^ cell isolates** | **Live cell count per isolate** (mean ± SD) |
| **1** | f | 49 | Mamma | 5 | 25.6 (± 3.1) ×10^6^ |
| **2** | f | 30 | Abdomen | 10 | 19.0 (± 5.5) ×10^6^ |
| **3** | f | 31 | Abdomen | 114 | 25.9 (± 11.2) ×10^6^ |
| **4** | f | 34 | Abdomen | 132 | 30.5 (± 13.3) ×10^6^ |
| **5** | f | 28 | Abdomen | 178 | 34.7 (± 16.5) ×10^6^ |
| **6** | f | 39 | Abdomen | 38 | 37.0 (± 15.0) ×10^6^ |
| **7** | f | 37 | Abdomen | 71 | 48.6 (± 16.9) ×10^6^ |

Included are all isolates that were pooled to the 47 drug substance batches released for drug production

*f* female

**Table S3** Results from drug substance batch release testing by donor

| **Parameter** | **Specification** | **Donor 1**  n = 1 | **Donor 2** n = 2 | **Donor 3** n = 14 | **Donor 4** n = 15 | **Donor 5** n = 19 | **Donor 6** n = 6 | **Donor 7** n = 9 |
| --- | --- | --- | --- | --- | --- | --- | --- | --- |
|  |  |  |  |  |  |  |  |  |
| ABCB5^+^ cell content | ≥ 90% | 95.20% | 96.50±0.35% | 96.18±2.49% | 98.58±0.85% | 97.60±1.49% | 99.27±0.58% | 98.31±1.69%^a)^ |
| Mycoplasma | not detectable (< 10 CFU/ml) | not detectable | not detectable in 2/2 batches | not detectable in 14/14 batches | not detectable in 15/15 batches | not detectable in 19/19 batches | not detectable in 5/6 batches | not detectable in 9/9 batches |
| Endotoxin level | ≤ 2 EU/ml | ≤ 2 EU/ml | ≤ 2 EU/ml in 2/2 batches | ≤ 2 EU/ml in 14/14 batches | ≤ 2 EU/ml in 15/15 batches | ≤ 2 EU/ml in 19/19 batches | ≤ 2 EU/ml in 6/6 batches | ≤ 2 EU/ml in 9/9 batches |
| Live cell count | n.a.^b)^ | 127.90 ×10^6^ | 94.75±10.39 ×10^6^ | 210.89±98.68 ×10^6^ | 268.41±113.69 ×10^6^ | 325.10±135.00 ×10^6^ | 234.38±132.82 ×10^6^ | 383.20±120.74 ×10^6^ |
| Deviation of live cell count from expected value^c)^ | ≤ 30% | n.a.^d)^ | n.a.^d)^ | n.a.^d)^ | ≤ 30% in 12/15 batches^e)^ | ≤ 30% in 19/19 batches | ≤ 30% in 6/6 batches | ≤ 30% in 9/9 batches |
| Cell vitality | ≥ 90% | 98.70% | 98.80±0.28% | 98.94±0.33% | 98.09±0.90% | 98.80±0.47% | 98.25±0.27% | 98.67±0.35% |
| Cell viability | ≥ 90% | 99.20% | 99.80±0.14% | 98.96±1.11% | 99.48±0.27% | 99.22±0.52% | 99.57±0.10% | 99.29±0.43% |
| CD90^+^ cell content | ≥ 90% | 99.40% | 99.90±0.14% | 99.44±0.53% | 99.41±0.34% | 99.50±0.61% | 99.67±0.12% | 99.30±0.71% |
| Bead residues | ≤ 0.5% | 0.02% | 0.00±0.00% | 0.02±0.03% | 0.02±0.05% | 0.04±0.03% | 0.09±0.06% | 0.09±0.05% |
| Microbiological control | no growth | no growth | no growth in 2/2 batches | no growth in 14/14 batches | no growth in 15/15 batches | no growth in 19/19 batches | no growth in 6/6 batches | no growth in 9/9 batches |
| IL‑1RA secretion | > 125 pg/ml | > 125 pg/ml | > 125 pg/ml in 2/2 batches | > 125 pg/ml in 14/14 batches | > 125 pg/ml in 15/15 batches | > 125 pg/ml in 19/19 batches | > 125 pg/ml in 6/6 batches | > 125 pg/ml in 9/9 batches |
| VEGF secretion | > 46.9 pg/ml | > 46.9 pg/ml | > 46.9 pg/ml in 2/2 batches | > 46.9 pg/ml in 14/14 batches | > 46.9 pg/ml in 15/15 batches | > 46.9 pg/ml in 19/19 batches | > 46.9 pg/ml in 6/6 batches | > 46.9 pg/ml in 9/9 batches |
| Angiogenic differentiation potential | Formation of capillary structures in one or both seeded cell concentrations | positive | positive in 2/2 batches | positive in 14/14 batches | positive in 13/15 batches | positive in 18/19 batches | positive in 6/6 batches | positive in 9/9 batches |

Numeric results are given as mean ± SD

*n.a.* not applicable

^a^For one batch of donor 7, measurement procedure was not fully GMP-conform. Although this did not affect the result, the batch was hold and eventually discarded

^b^Acceptance criteria for cell count are only defined for the for the aliquots into which the drug substance batches are divided for cryostorage, not for the master batch as a whole

^c^Expected value = sum of the live cell counts of all ABCB5^+^ cell isolates that were pooled to produce the drug substance batch

^d^The criterion “Deviation of live cell count from expected value” was implemented only from donor 4 on

^e^Deviations were most likely caused by inhomogeneous distribution of the cells during filling into cryovials. As a consequence, additional mixing steps were implemented in the filling process
