# Supplementary material for: Process data of allogeneic ex vivo-expanded ABCB5+ mesenchymal stromal cells for human use: off-the-shelf GMP-manufactured donor-independent ATMP
Source: Stem Cell Res Ther. 2020 Nov 16;11:482. doi: 10.1186/s13287-020-01987-y (PMC7667860; doi:10.1186/s13287-020-01987-y)
Supplement: Supplementary file 2 — Additional file 2 : Fig. S1. Photographic documentation of the cell monolayer at 70% confluency. Original total magnification × 200. Fig. S2. Tube formation assay on extracellular matrix gel. ABCB5+ mesenchymal stem cells (1 × 10^5^ and 1.5 × 10^5^) were seeded in two wells of a Geltrex™-coated 24-well plate and incubated for 19–22 h. Tube formation was evaluated visually according to the following categories: A tubular branches of several cells forming a defined network-like structure; B tubular branches of several cells clustering together forming broad strands, formation of syncytia, areas of high cellular density lacking formation of tubular branches; C cells clustering together, building nodes and forming tubular branches that connect the nodes with each other; D only sporadic cells form tubular branches, partial node formation, but no or nearly no connections between nodes, no or only sporadic apoptotic cells; E largely apoptotic cells, no or only sporadic tubular branches; F no tubular branches. Human umbilical vein endothelial cells (HUVEC) and human skin melanoma cells (SK-MEL-28) served as positive and negative controls, respectively. Categories A–C are considered as successful angiogenic differentiation. Original total magnification × 40. [file 13287_2020_1987_MOESM2_ESM.pptx]

## Slide 1
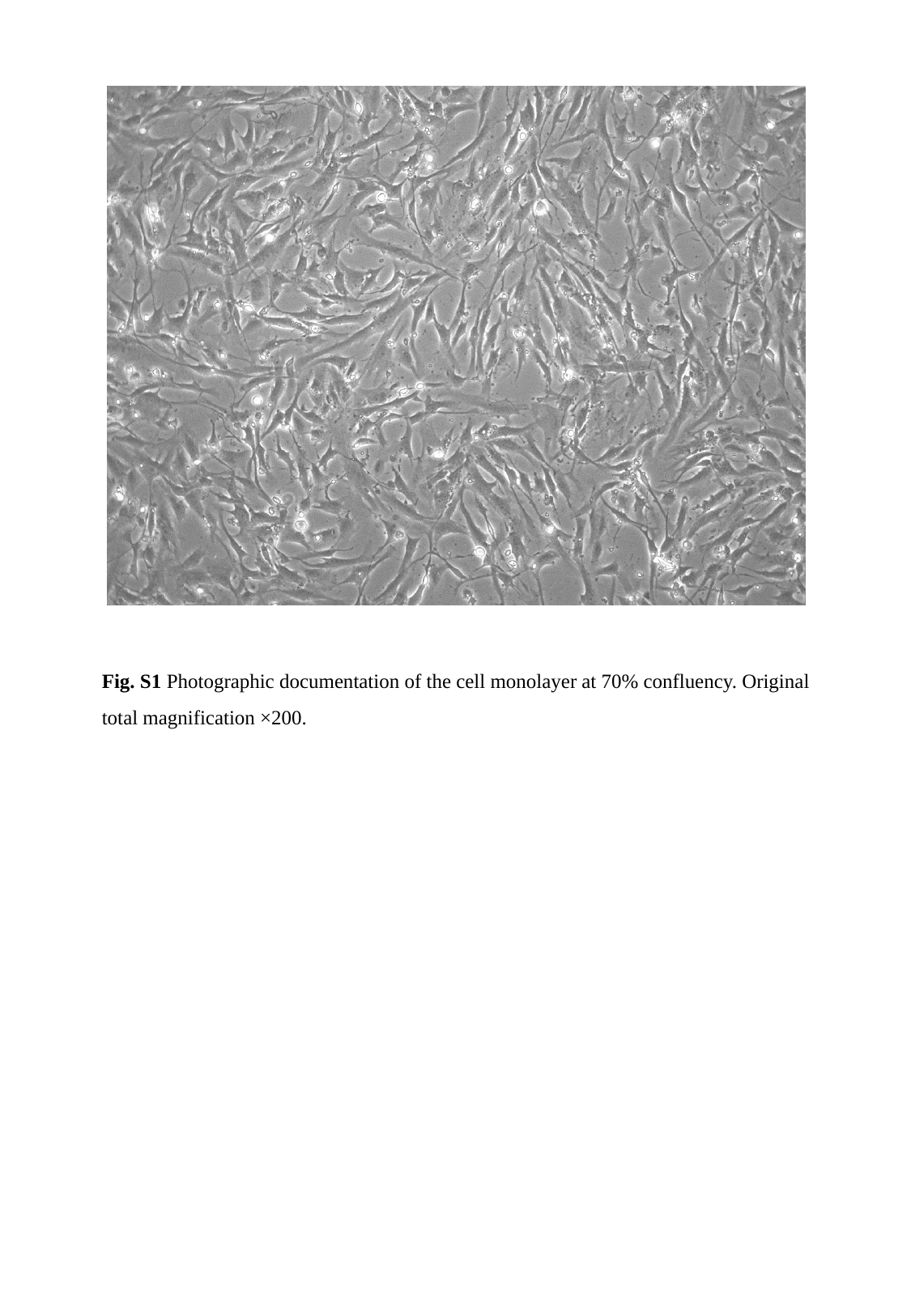

Fig. S1 Photographic documentation of the cell monolayer at 70% confluency. Original total magnification ×200.

## Slide 2
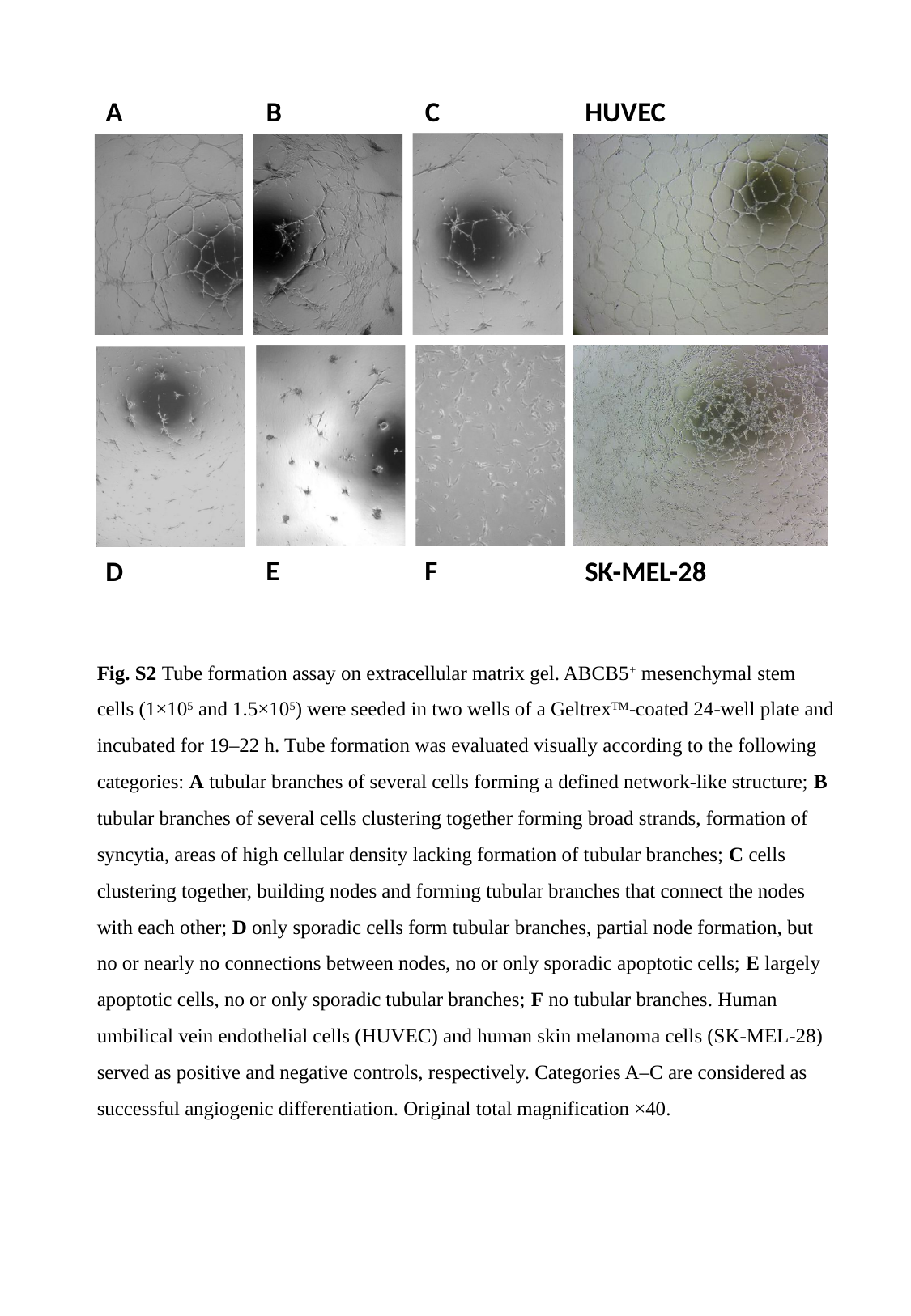

C
B
A
HUVEC
F
E
D
SK-MEL-28
Fig. S2 Tube formation assay on extracellular matrix gel. ABCB5+ mesenchymal stem cells (1×10^5^ and 1.5×10^5^) were seeded in two wells of a GeltrexTM-coated 24‑well plate and incubated for 19–22 h. Tube formation was evaluated visually according to the following categories: A tubular branches of several cells forming a defined network-like structure; B tubular branches of several cells clustering together forming broad strands, formation of syncytia, areas of high cellular density lacking formation of tubular branches; C cells clustering together, building nodes and forming tubular branches that connect the nodes with each other; D only sporadic cells form tubular branches, partial node formation, but no or nearly no connections between nodes, no or only sporadic apoptotic cells; E largely apoptotic cells, no or only sporadic tubular branches; F no tubular branches. Human umbilical vein endothelial cells (HUVEC) and human skin melanoma cells (SK‑MEL‑28) served as positive and negative controls, respectively. Categories A–C are considered as successful angiogenic differentiation. Original total magnification ×40.
